# Thymic stromal lymphopoietin in leukemia: A double-edged sword?

**DOI:** 10.1016/j.lrr.2025.100530

**Published:** 2025-07-23

**Authors:** Xing Zou, Mengmeng Gu, Yue Su, Dayong Yao, Hao Gang, Yang Li, Ce Shi

**Affiliations:** a NHC Key Laboratory of Cell Transplantation, The First Affiliated Hospital of Harbin Medical University, Harbin, China; b Department of Urology, The First Affiliated Hospital of Harbin Medical University, Harbin, China; c Harbin Medical University, Harbin, China

**Keywords:** TSLP, Leukemia, Dual mechanism of TSLP

## Abstract

Thymic stromal lymphopoietin (TSLP) is a cytokine expressed by various cell types. It is involved in the development of allergies, autoimmune diseases, and cancers. TSLP affects immune responses and disease pathogenesis by binding to a heterodimeric receptor complex. In leukemia, particularly acute lymphoblastic leukemia (ALL), the importance of TSLP is being increasingly recognized. It has been shown that TSLP promotes leukemia proliferation by regulating different signaling pathways. However, it has also shown that other gene mutations can occur during this proliferative process, making it impossible to completely cure leukemia by targeting TSLP alone. Additionally, effect of TSLP on leukemia was found to be dose dependent. Whilst low doses were found to support the proliferation of leukemia cells, high doses were found to induce apoptosis in leukemia cells. This review focuses on the dual mechanisms of TSLP in leukemia, providing new insights for leukemia treatment.

## Highlights

• **First demonstration of TSLP's dual roles in leukemia:** TSLP promotes proliferation via CRLF2 signaling while inducing apoptosis in specific contexts, revealing its paradoxical tumor-modulating effects.
• **Elucidates biphasic TSLP regulation:** Dose-dependent signaling thresholds determine the proliferation-apoptosis switch, redefining therapeutic windows.
• **Proposes precision medicine approach:** Genetic biomarker-guided TSLP targeting addresses response heterogeneity, enabling personalized treatment strategies.

## Graphical abstract

Dual Mechanisms of TSLP in Leukemia. This figure was created using Figdraw.

**Figure:**
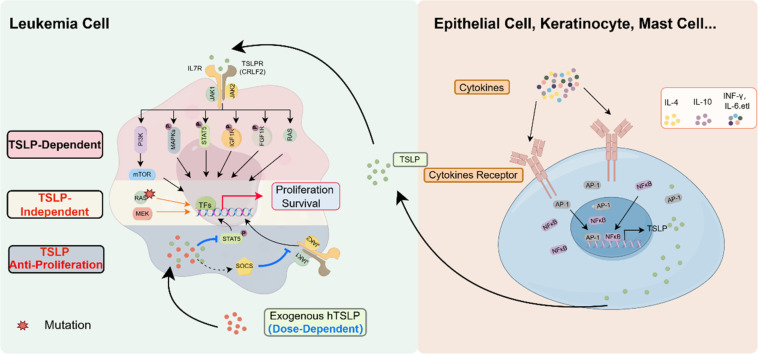

## 1 TSLP

### 1.1 Introduction

Thymic stromal lymphopoietin (TSLP) is an IL-7-like cytokine initially identified in the supernatant of cultured murine thymic stromal cell lines [1]. Murine TSLP is a protein composed of 140 amino acids, it located on chromosome 18. The Human TSLP protein exists in two isoforms, one with a length of 159 amino acids and the other comprising 63 amino acids, with its encoding gene located at 5q22.1 near the atopy cytokine cluster on chromosome 5q31 [2,3]. TSLP is primarily produced by epithelial cells (ECs) and keratinocytes (KCs) [[4], [5], [6], [7]]. Some of the other cells expressing TSLP, include mast cells [8], smooth muscle cells [9], cancer-associated fibroblasts [10], dendritic cells (DCs), and trophoblasts [11].

TSLP production is stimulated in response to various stimuli, such as bacteria [[12], [13], [14]], viruses [[15], [16], [17]], Toll-like receptor (TLR) ligands [6,[18], [19], [20]], worms [21], and allergens [22]. *In-vivo*, cytokines are also found to be important triggers for increased TSLP -production and -secretion [4,5]. The expression of TSLP is tightly regulated by both transcriptional and post-transcriptional mechanisms. Transcriptionally, Cytokines play a significant role in the production of TSLP. For example, proinflammatory cytokines (such as TNF-α or IL-1α), Th2 cytokines (like IL-4 or IL-13), as well as IFN-γ and TGF-β, can induce TSLP production [5,23,24].TSLP is regulated by key transcription factors like NF-κB, and AP-1 [25], which upregulate its transcription. Nuclear receptors such as the retinoid X receptor (RXR), vitamin D receptor (VDR), and glucocorticoid receptor (GR) also play crucial roles in TSLP regulation. Ablation of RXR in epidermal keratinocytes [26] or treatment with VDR ligands [27] such as calcitriol leads to increased TSLP production in the skin. In human bronchial epithelial cells, both the RXR agonist 9-cis-retinoic acid and glucocorticoids acting through GR inhibit TSLP expression [28]. Post-transcriptionally, TSLP expression is influenced by microRNAs (miRNAs) that modulate mRNA stability and thus translation [[29], [30], [31]].

TSLP supports the development of pro-B cells into B220 + IgM+ immature B cells [32,33]. *In-vivo* it can activate pro-allergic CD4+ and CD8+ *T* cells [34,35], inducing Th2-type hypersensitivity reactions. At barrier surfaces, such as the skin, lungs, and intestines, TSLP can trigger allergic diseases such as atopic dermatitis [36,37] and bronchial asthma [38,39]. Increasing evidence suggests that TSLP is also closely associated with the development of autoimmune diseases [[40], [41], [42]] and cancers [[43], [44], [45], [46], [47]].

### 1.2 The mechanism of action of TSLP

TSLP exerts its effects through binding to a heterodimeric receptor complex consisting of the TSLP receptor (TSLPR) and the interleukin-7 receptor alpha chain (IL-7Rα) [48,49]. TSLPR and IL-7Rα are critical for TSLP binding. TSLPR is encoded by the cytokine receptor like factor2 (CRLF2) gene. IL-7Rα, also known as CD127, is a component shared with the interleukin-7 (IL-7) receptor. It is encoded by the IL7R gene located on chromosome 5.

The binding of TSLP to its receptor complex begins with TSLP initially interacting with TSLPR. This interaction causes a conformational change in TSLPR that facilitates the recruitment of IL-7Rα to form a high-affinity heterodimeric receptor complex. Once the heterodimeric complex is formed, it induces further conformational changes that are crucial for the activation of downstream signaling pathways [50].

### 1.3 TSLP in diseases

TSLP plays a critical role in various diseases, including asthma [38,39], atopic dermatitis [36,37], inflammatory bowel diseases [51], coronary artery disease [52,53], and certain cancers [[44], [45], [46], [47], [48]]. It also regulates immune responses by promoting Th2 inflammation, activating dendritic and T cells, and enhancing cytokine production, contributing to disease pathogenesis [54].

TSLP exhibits diverse roles in various diseases, including contradictory effects in cancers such as breast cancer. Some studies suggest that TSLP can promote the growth and metastasis of breast cancer, indicating a pro-tumorigenic role [44,55]. Contrastingly, other study shows that high expression of TSLP in keratinocytes within mouse models can suppress breast cancer development, suggesting a protective effect [56]. Furthermore, the study using human breast cancer cell lines also indicate that TSLP signaling might not be a critical pathway in the progression of human breast cancer [57]. These conflicting findings underscore the complexity of TSLP's role in cancer biology and highlight the need for more research to clarify its mechanisms and potential as a therapeutic target.

## 2 TSLP in leukemia

Leukemia is a malignant blood disease characterized by abnormal proliferation of white blood cells. Despite advancements in treatment modalities, leukemia remains a leading cause of cancer-related death in children, particularly in cases of treatment failure and relapse [58]. In children, acute lymphoblastic leukemia (ALL) accounts for nearly 80 % of all leukemia cases [59]. ALL is the malignant transformation and proliferation of lymphoid progenitor cells in the bone marrow, blood, and extramedullary sites. ALL can be classified into B-lineage or T-lineage lymphoid progenitor cells based on their origin [60].

Astrakhan et al. observed that overexpression of TSLP in the early postnatal period is sufficient to drive B cell lymphoproliferative disorders [61]. This suggests that TSLP's impact on leukemia, particularly ALL, may be significantly influenced by the timing of its expression. Specifically, while early postnatal TSLP overexpression can promote the development of B-cell disorders, administering or inducing TSLP after day 14 post-birth does not produce the same effect [61]. This phenomenon highlights that TSLP's role in leukemia might vary depending on the developmental stage.

Similarly, the regulatory role of TSLP in ALL is also controversial, with some studies suggesting that it has a bidirectional regulatory effect. The current review aims to focus on the effects of TSLP in different types of ALL and the mechanisms underlying its bidirectional regulation.

### 2.1 Positive regulation of leukemia proliferation by TSLP

#### 2.1.1 TSLP is associated with poor prognosis in leukemia

##### 2.1.1.1 B-ALL

A study of pediatric patients with B-cell precursor acute lymphoblastic leukemia (BCP-ALL) identified mutations in the TSLP receptor CRLF2 with the highest frequency in 45 patients accounting for 14.3 % of all cases [62].

Studies have assessed CRLF2 expression in various cohorts of pediatric and adult patients with B-cell acute lymphoblastic leukemia (B-ALL) and found significant prognostic associations [[63], [64], [65]]. In a study involving bone marrow samples from 117 newly diagnosed pediatric BCP-ALL patients, high CRLF2 expression was found to be associated with significantly worse event-free survival (EFS) as compared to patients with low CRLF2 expression [63]. Another study demonstrated that poor outcomes in pediatric B-cell acute lymphoblastic leukemia are associated with high levels of CRLF2 gene expression across distinct molecular subtypes [64]. In adult patients with B-cell precursor acute lymphoblastic leukemia, high CRLF2 expression was also associated with higher white blood cell (WBC) counts, the presence of P2RY8-CRLF2 fusion, and IKZF1 deletions (IKZF1del) [65]. These factors collectively contribute to a poorer prognosis.

Compared to non-Philadelphia-like patients, those with Philadelphia-like ALL (Ph-like ALL) exhibit lower rates of complete remission (CR), EFS, and disease-free survival (DFS). Overexpression of the CRLF2 is present in 35.7 % of Ph-like ALL cases, which contributes to poor treatment outcomes [66]. In 91 % of Ph-like ALL patients, rearrangements involving various kinases, including CRLF2, have been identified [67]. These findings underscore the importance of CRLF2 expression levels as a prognostic marker in both pediatric and adult B-ALL.

CRLF2 rearrangements are overexpressed in approximately 50–60 % of Down syndrome-associated acute lymphoblastic leukemia (DS-ALL) cases [68,69]. Notably, most DS-ALL cases are classified as B-ALL [70]. DS-ALL is typically resistant to treatment and is associated with a poor prognosis [71]. Studies have reported that High-mobility group nucleosome binding domain 1 (HMGN1) plays a significant role in CRLF2-driven Down syndrome leukemia [72], providing a potential therapeutic target in this high-risk cohort.

##### 2.1.1.2 T-ALL

Research on CRLF2 alterations in T-ALL is limited, but emerging evidence suggests its role as a poor prognostic marker in high-risk cases. Approximately 10 % of T-ALL cases exhibit somatic mutations in IL7Rα [73,74]. Also, CRLF2 overexpression is identified as a significant adverse prognostic factor in these patients.

A Chinese study analyzing CRLF2 expression in 218 newly diagnosed pediatric ALL patients found that high CRLF2 expression is associated with significantly higher relapse rates and poorer EFS [75]. Similarly, an analysis of CRLF2 expression in 212 pediatric T-ALL patients from the Italian and German AIEOP-BFM ALL 2000 cohort revealed that 17 out of 120 Italian patients (14.2 %) had CRLF2 mRNA expression levels five times higher than the median [76]. These patients demonstrated significantly lower EFS, and overall survival (OS) rates compared to those with low CRLF2 expression. Moreover, the cumulative incidence of relapse and resistance was significantly higher in the high-CRLF2 group. Notably, CRLF2-high samples displayed stronger TSLP-induced signal transducer and activation of transcription (STAT5) phosphorylation, which could be completely inhibited by the Janus kinase (JAK) inhibitor ruxolitinib.

CRLF2 overexpression in T-ALL has been linked to specific molecular alterations, such as mutations in PTEN, JAK3, PHF6, EZH2, and RUNX1, as well as TCF7 deletions [77]. These genetic disruptions are associated with increased CRLF2 transcription levels, contributing to the aggressive nature of the disease. Additionally, CRLF2 overexpression has been shown to correlate with ICN1 stabilization in T-ALL, further driving leukemogenesis [78].

Recent studies have identified that BET inhibition can reverse CRLF2 overexpression in early T-cell precursor acute lymphoblastic leukemia (ETP-ALL) cell lines, suggesting a link with PRC2 dysfunction [77]. Restoration of PRC2 function was found to mitigate CRLF2 overexpression, indicating a potential therapeutic target for treating high-risk T-ALL patients.

CRLF2 overexpression serves as a critical prognostic marker in pediatric ALL, particularly in B-ALL and high-risk T-ALL subtypes. Its presence is associated with poor clinical outcomes, including lower EFS and OS rates, and higher relapse and resistance rates. This association underscores the importance of exploring the mechanisms through which TSLP promotes proliferation and the potential therapeutic interventions targeting CRLF2 and related pathways.

#### 2.1.2 Pathways promoting leukemia proliferation by TSLP

##### 2.1.2.1 TSLP/TSLPR-JAK-STAT

TSLP promotes leukemia cell proliferation primarily through its receptor (TSLPR), encoded by the CRLF2 gene, and the downstream JAK-STAT signaling pathway. This pathway is notably hyperactive in cases of B-ALL with CRLF2 rearrangements [79]. Previous study was found that approximately half of CRLF2-rearranged BCR-ABL1-like B-ALL cases exhibited concomitant activating mutations in JAK2, whereas such mutations were rare in non-CRLF2-rearranged cases [80]. These mutations contribute to the aggressiveness and poor prognosis of the leukemia.

The study observed that in cells overexpressing CRLF2 F232C, genes in the JAK/STAT pathway were upregulated. Cells with CRLF2 aberrations demonstrated increased resistance to cyclophosphamide and other commonly used chemotherapeutic agents [81]. Recent studies have also indicated that CRLF2 is regulated epigenetically. Specifically, the inherited genetic variant rs3824662 functions as a *cis*-acting enhancer variant that activates GATA3 transcription [82]. This alteration in global chromatin status plays a role in the pathogenesis of Ph-like ALL by regulating CRLF2 signaling.

##### 2.1.2.2 PI3K/mTOR

Mammalian target of rapamycin (mTOR) is a serine/threonine kinase that functions as a central regulator of cell growth and division [83,84]. It induces cell cycle progression, cap-dependent translation, and ribosome synthesis. As a sensor, it ensures cells are in an adequate -nutritional and -energetic state before they grow and proliferate. Dysregulation and constitutive activation of the phosphatidyl inositol 3 kinase (PI3K/mTOR) pathway have been implicated in the proliferation and survival of various cancers [85].

In a mouse model of Down syndrome and in DS-ALL patient samples, CRLF2 overexpression leads to reduced B-cell differentiation and enhanced E2F signaling. This enhancement is mediated through the activities of the PI3K/mTOR pathway and CDKs [86]. The PI3K/mTOR inhibitor AZD2014 and the pan-CDK inhibitor AT7519 exhibited significant cytotoxic effects in CRLF2+ ALL cells. Flow cytometry and Western blotting analyses revealed alterations in cell proliferation and pathway activation, underscoring the pivotal role of the PI3K/mTOR pathway in these malignancies.

Additional studies demonstrated that TSLP stimulation of CRLF2-rearranged leukemia cells resulted in aberrant STAT5 and PI3K/mTOR pathway signaling [87]. Furthermore, a novel xenograft model using primary CRLF2 B-ALL cells in immunodeficient mice treated with human TSLP (hTSLP) showed induction of mTOR-regulated genes. RNA sequencing (RNA-seq) and quantitative PCR (qPCR) analyses of the xenograft tumors confirmed the expression of mTOR-regulated genes in primary CRLF2 B-ALL cells [88], substantiating the involvement of the PI3K/mTOR pathway in TSLP/TSLPR-mediated leukemia proliferation.

These findings highlight the integral role of the PI3K/mTOR pathway in the pathogenesis of Ph-like ALL and propose this pathway as a potential therapeutic target.

##### 2.1.2.3 MAPK

TSLP stimulation resulted in the phosphorylation of several kinases including Lyn, Btk, Hck, Syk, MAPK8, MAPK9, and MAPK10, all of which are integral components of the MAPK pathway [89]. These kinases activate downstream proliferation-related pathways, highlighting the critical role of MAPK signaling in TSLP-mediated leukemia proliferation. However, in cells dependent on CRLF2 or mutated JAK2, the study observed reduced phosphorylation at these targets, suggesting distinct signaling dependencies based on mutation status.

##### 2.1.2.4 Other factors influencing TSLP-Mediated leukemia proliferation

Recent studies have elucidated the diverse signaling mechanisms activated by TSLP in leukemia cells, with a particular focus on CRLF2-overexpressing Ph-like ALL cell lines. Upon TSLP stimulation of these cell lines followed by quantitative phosphotyrosine (P-Tyr) analysis, previously unrecognized tyrosine kinases and signaling pathways were identified, including the insulin-like growth factor 1 receptor (IGF1R) and fibroblast growth factor receptor 1 (FGFR1) proteins [90], both upregulated by TSLP. The activation of IGF1R and FGFR1 presents a novel mechanism through which TSLP may promote cellular proliferation.

Additionally, TSLP has been shown to induce RAS activity in the MUTZ5 cell line and in DS-ALL patient samples [47]. This significant finding indicates that the activation of the RAS pathway can occur independently of RAS mutation status. It also suggests that RAS protein activation may serve as a potential predictive marker and therapeutic target for high-risk ALL.

Collectively, these studies reveal that TSLP stimulation activates a broad spectrum of signaling pathways through tyrosine phosphorylation, affecting proteins and pathways beyond the previously established TSLP/CRLF2 axis. The identification of IGF1R and FGFR1 as novel components of TSLP/CRLF2 signaling enhances our understanding of the molecular mechanisms that underlie TSLP-mediated leukemia proliferation.

#### 2.1.3 Targeting TSLP for leukemia treatment

Targeting TSLP and its receptor shows significant potential in the treatment of ALL. CRLF2 is commonly overexpressed in B-ALL and is associated with poor prognosis and high relapse rates. By targeting TSLP and its receptor, key cellular signaling pathways can be disrupted, inhibiting leukemia cell growth and spread, thus providing new therapeutic options and hope for high-risk leukemia patients.

Targeting TSLP and its receptor holds significant promise for the treatment of ALL. The CRLF2 is commonly overexpressed in B-ALL and is linked to poor prognosis and high relapse rates. By inhibiting TSLP and its receptor, crucial cellular signaling pathways can be disrupted. This disruption can inhibit the growth and spread of leukemia cells, offering new therapeutic options and renewed hope for high-risk leukemia patients.

Blocking the TSLP receptor significantly inhibits TSLP-driven proliferation and signaling in lymphoblasts from subsets of B-precursor ALL patients [91]. Studies have demonstrated that TSLP receptor blockade effectively reduces leukemia cell growth and suppresses associated signaling pathways. Chimeric antigen receptor (CAR) T-cell therapy targeting TSLPR has shown efficacy in CRLF2-rearranged B-ALL. These CAR T-cells specifically recognize and kill TSLPR-expressing leukemia cells [92]. In several xenograft models, this therapy has demonstrated significant anti-leukemia activity and offers promising treatment options for high-risk leukemia patients.

Research indicates that combining signal transduction inhibitors (such as JAK inhibitors) with direct targeting of the TSLP receptor can be an effective treatment strategy for DS-ALL [93]. These combination therapies significantly inhibit the JAK/STAT signaling pathways, thus reducing leukemia cell proliferation. Notably, combining ruxolitinib with standard treatment drugs has shown significant therapeutic effects in CRLF2-rearranged Ph-like ALL acute lymphoblastic leukemia [94]. This combination therapy enhances the suppression of leukemia cells, thereby improving treatment efficacy.

Recombinant antibody fragments directed against the human TSLPRα chain extracellular domain have shown significant antagonistic effects on STAT5 signaling. These antibodies effectively target Ph-like ALL by blocking TSLPR function, thereby inhibiting leukemia cell proliferation [95]. The 1B7/CD3 is a novel bispecific antibody targeting CRLF2-rearranged Ph-like B-ALL. This antibody simultaneously targets TSLPR and CD3, activating T cells to induce leukemia cell killing. Preclinical studies have shown that 1B7/CD3 exhibits significant anti-tumor activity in both *in vitro* and *in vivo* models [96].

Various therapeutic strategies targeting TSLP and its receptor show significant potential in the treatment of ALL. Approaches such as monoclonal antibodies, bispecific antibodies, CAR T-cells, and combined signal transduction inhibitors can effectively inhibit leukemia cell proliferation and survival, offering new therapeutic hope for high-risk leukemia patients.

### 2.2 Leukemia proliferation is TSLP-independent or exhibits opposing effects

Although targeting TSLPR has proven to be an effective strategy for inhibiting leukemia cell proliferation, some studies suggest that TSLP might not play a significant role in promoting leukemia cell growth and may even have opposing effects. These studies highlight TSLP-independent mechanisms in leukemia cell proliferation, providing new insights for clinical treatment. Recent research has shown that ruxolitinib treatment fails to inhibit the growth of CRLF2-rearranged cells, despite significantly reducing pSTAT5 levels [97]. Additionally, CAR-T therapy has been demonstrated to significantly reduce CRLF2 expression in the spleen and blood of CRLF2-rearranged PDX mouse models, but it does not significantly clear CRLF2 expression in the bone marrow [92]. Furthermore, in relapsed CRLF2-rearranged Patient-Derived Xenograft (PDX) mouse models, CAR-T therapy still leaves CRLF2 on the cell surface. This indicates that directly targeting TSLP alone may be insufficient to completely cure leukemia, possibly because TSLP is non-essential for leukemia cell proliferation.

#### 2.2.1 Independent TSLP mutations

The activation of the TSLP/TSLPR-JAK signaling pathway is considered a crucial regulatory mechanism in the development of Ph-like ALL. However, increasing research reveals that the use of JAK inhibitors alone does not inhibit the proliferation of Ph-like ALL cells. This suggests the presence of other genetic mutations driving the persistent proliferation of leukemia cells. One study conducted a genome-wide CRISPR-Cas9 screening on IgH—CRLF2-r ALL cell lines in the presence or absence of ruxolitinib, discovering that RAS mutations and/or mTORC1 signaling activation render cells insensitive to ruxolitinib [98]. When gilteritinib was used in combination with the MEK1/2 inhibitor trametinib, it effectively blocked the RAS and mTORC1 pathways, exhibiting significant anti-leukemia activity.

Glucocorticoids (GCs) are used clinically to treat ALL patients, but studies suggest that GC resistance may be associated with aberrant signaling related to CRLF2 overexpression [99]. When Ph-like ALL cells from patient-derived xenografts were exposed to GCs, CRLF2-rearranged leukemia consistently exhibited reduced GC sensitivity in vitro. However, targeting signal transduction with the MEK inhibitor trametinib and the Akt inhibitor MK2206, instead of the JAK inhibitor ruxolitinib, enhanced GC sensitivity in CRLF2-rearranged cells.

Although targeting TSLPR has shown efficacy to some extent, TSLP-independent mechanisms play a crucial role in leukemia proliferation. Therefore, a combination of multiple signal pathway inhibitors might be the key to improving therapeutic outcomes for leukemia in the future.

#### 2.2.2 Dose-dependent effects of TSLP on leukemia

Previous research has demonstrated that TSLP can have bidirectional effects in solid tumors, such as breast [44,55,56] and skin cancers [[100], [101], [102]]. Recent studies have suggested that high-dose TSLP can induce apoptosis in CRLF2 B-ALL cells [103]. In CRLF2 B-ALL, TSLP plays a dual role in leukemia cell proliferation, closely related to its dosage. Specifically, high-dose TSLP (10 ng/mL) supports the survival of normal B-cell precursors, whereas higher doses of TSLP (greater than 200 pg/mL) can induce apoptosis in CRLF2 B-ALL cells [104]. Similarly, high-dose TSLP (100–200 ng/mL) induces apoptosis in colon cancer cells in vitro and reduces tumor size when injected peritumorally [105]. Conversely, low-dose TSLP (approximately 20 pg/mL) supports the survival of CRLF2 B-ALL cells [88]. Given that the normal physiological levels of TSLP in children (13–32 pg/mL) are relatively low [106], it is suggested that during the development of leukemia, TSLP primarily promotes tumor cell proliferation at low levels. Thus, administering high levels of TSLP may offer a novel therapeutic strategy for treating CRLF2 B-ALL, providing new insights into clinical leukemia treatment. TSLP also exhibits contrasting effects on the progression of breast and skin cancers, although these effects have not been clearly linked to dosage. Therefore, the mechanisms by which TSLP exerts its dual effects vary among different tumor types.

## 3 Conclusion and outlook

TSLP plays a critical role in various diseases, including asthma [38,39], atopic dermatitis [36,37], inflammatory bowel disease [51], coronary artery disease [52,53], and certain cancers [[43], [44], [45], [46], [47]]. Primarily produced by epithelial cells and keratinocytes [[4], [5], [6], [7]], TSLP exerts its effects by binding to its receptor complex, which consists of TSLPR and IL-7Rα, thereby activating downstream signaling pathways [[48], [49], [50]]. Its involvement is particularly notable in conditions such as asthma, atopic dermatitis, and specific cancers, where it drives T helper 2 (Th2) inflammation and immune responses [54].

In the context of leukemia, particularly ALL, TSLP exhibits a complex dual role. High expression levels of CRLF2 in both B-cell acute lymphoblastic leukemia (B-ALL) [[62], [63], [64], [65], [66], [67]] and T-cell acute lymphoblastic leukemia (T-ALL) [[73], [74], [75], [76]] are often correlated with poor prognosis and resistance to treatment. TSLP may promote leukemia cell proliferation through several signaling pathways, including JAK-STAT [[79], [80], [81], [82]], PI3K/mTOR [[83], [84], [85], [86], [87], [88]], and MAPK [89], as well as through other factors such as RAS [47], IGF1R, and FGFR1 [90]. Targeting TSLP and its receptor using approaches such as CAR T-cell therapy [90,92] and JAK inhibitors [63] has emerged as a promising strategy for treating high-risk leukemia patients. However, some studies indicate that TSLP may not be essential for leukemia proliferation [98,99], with high concentrations of TSLP potentially inducing apoptosis in CRLF2 B-ALL cells [104].

In conclusion, the dual role of TSLP in CRLF2 B-ALL highlights the complexities involved in its use as a therapeutic target and presents new opportunities for future treatment strategies. By meticulously investigating the relationship between TSLP dosage and its biological effects, we can establish a robust foundation for the precise treatment of leukemia. The mechanisms by which high-dose TSLP induces tumor cell apoptosis have not been comprehensively studied. Future research should focus on elucidating the molecular pathways involved in the pro-tumor and anti-tumor effects of TSLP. Additionally, considering the individual TSLP levels and leukemia subtypes in patients will be crucial in selecting appropriate therapeutic approaches to further enhance treatment efficacy.

## Ethical approval

Ethical approval is not applicable to this article.

## CRediT authorship contribution statement

**Xing Zou:** Investigation, Writing – original draft, Visualization. **Mengmeng Gu:** Investigation, Writing – original draft, Visualization. **Yue Su:** Conceptualization, Investigation. **Dayong Yao:** Investigation, Conceptualization. **Hao Gang:** Conceptualization. **Yang Li:** Writing – review & editing. **Ce Shi:** Writing – review & editing, Funding acquisition.

## Declaration of competing interest

The authors declare no conflicts of interest.

## Data Availability

Data availability is not applicable to this article.
